# The Current Perspective, Ethical Considerations, and a Position Statement on Expanded Newborn Screening from the College of Paediatrics, Academy of Medicine of Malaysia

**DOI:** 10.3390/ijns12040079

**Published:** 2026-09-30

**Authors:** Erwin Jiayuan Khoo, Meow Keong Thong, Sin Chuen Yong, Seok Chiong Chee, Jimmy Kok Foo Lee, Siao Hean Teh, Fahisham Taib, Adli Ali, Fook Choe Cheah

**Affiliations:** 1 Department of Paediatrics, IMU University, Kuala Lumpur 57000, Malaysia; jiayuan_khoo@imu.edu.my; 2 Center for Bioethics, Harvard Medical School, Boston, MA 02115, USA; 3 M. Kandiah Faculty of Medicine and Health Sciences, Universiti Tunku Abdul Rahman, Kajang 43000, Selangor, Malaysia; thongmk@utar.edu.my; 4 Genetic Medicine Division, Universiti Malaya Medical Centre, Kuala Lumpur 59100, Malaysia; 5 Department of Paediatrics, Pantai Hospital Kuala Lumpur, Kuala Lumpur 59100, Malaysia; scyong888@yahoo.co.uk; 6 School of Medicine, Faculty of Health and Medical Sciences, Taylor’s University, Subang Jaya 47500, Selangor, Malaysia; seokchiong@gmail.com; 7 Jeffrey Cheah School of Medicine & Health Sciences, Monash University Malaysia, Clinical School Johor Bahru, Johor Bahru 80100, Johor, Malaysia; leekokfoo@yahoo.com; 8 Department of Paediatrics, Sarawak General Hospital, Kuching 93586, Sarawak, Malaysia; tehshean@gmail.com; 9 Department of Paediatrics, School of Medical Sciences, Health Campus, Universiti Sains Malaysia, Kubang Kerian 16150, Kelantan, Malaysia; fahisham@usm.my; 10 Department of Paediatrics, Faculty of Medicine, Universiti Kebangsaan Malaysia, Kuala Lumpur 56000, Malaysia; adli.ali@hctm.ukm.edu.my; 11 Research Centre, Hospital Tunku Ampuan Besar Tuanku Aishah Rohani, Kuala Lumpur 56000, Malaysia; 12 Jeffrey Cheah Sunway Medical School, Faculty of Medical and Life Sciences, Sunway University, Bandar Sunway, Subang Jaya 47500, Selangor, Malaysia

**Keywords:** equity, expanded, genetic testing, justice, sustainable, tandem mass spectrometry

## Abstract

Newborn screening (NBS) is an essential public health intervention for early detection and secondary prevention of inherited disorders in asymptomatic infants at birth. Advances in this field and the accessibility of new screening methods and therapies in the last decade have insurmountably surpassed Malaysia’s national NBS programme, which has stagnated. Currently, our mandatory nationwide routine screening is limited to only two conditions: glucose-6-phosphate dehydrogenase deficiency and congenital hypothyroidism. While services such as universal newborn hearing and critical congenital heart disease screening are available in many settings, most treatable genetic and metabolic disorders remain unscreened. Consequently, preventable morbidity and mortality, from inborn errors of immunity and metabolism, and genetic diseases persist. This position statement reviews the evidence for expanding the national NBS programme in Malaysia, arguing that a modernised, contemporary and pragmatic method is both clinically tenable and morally imperative. By embracing advancements for expanded NBS, Malaysia can safeguard children’s right to an open future, fulfil intergenerational justice, and ensure that no infant is left without the opportunity for life-saving care.

## 1. Introduction

Newborn screening (NBS) is one of the most successful public health interventions of the twentieth century, transforming neonatal care through the principle that early identification of treatable conditions yields better outcomes than delayed diagnosis [1]. In Malaysia, universal NBS currently includes glucose-6-phosphate dehydrogenase (G6PD) deficiency and congenital hypothyroidism (CH), both of which have substantially reduced preventable morbidity and mortality. Malaysia prides itself as a leader in NBS as the country became one of the first in the world to introduce nationwide NBS for G6PD deficiency. However, progress has stagnated despite major advances in molecular diagnostics and genomic medicine; the national NBS programme has not expanded in parallel with international recommendations and practice. While this position statement acknowledges the importance of other established neonatal screening and surveillance programmes, such as universal newborn hearing, critical congenital heart disease, developmental dysplasia of the hip, retinopathy of prematurity, and routine neonatal physical examinations, its focus is specifically on laboratory-based screening for metabolic, endocrine, immunological, genetic, and other conditions detectable through population-level newborn blood spot testing. The absence of universal screening for many of these treatable disorders continues to result in avoidable mortality, irreversible disability, and substantial long-term healthcare costs.

The burden of a limited screening program is impossible to ignore. Data from the Ministry of Health’s Children Statistics, Malaysia (2022), showed that congenital conditions remained the second most common principal cause of death for children under five in 2021, at 29.9 per cent [2]. Data from selective screening studies in Malaysia reveal a mortality rate of 1.4 per 10,000 children under-five attributed to inborn errors of metabolism (IEM) [3], with most deaths occurring in the neonatal period. The window of opportunity for early detection and effective intervention is shortly after birth to deliver the greatest impact in preventing poor outcomes. The current expanded NBS programme recommends a panel of over 30 to 50 conditions to be screened, the majority of which are IEMs. Other inherited conditions are inborn errors of immunity (IEIs), such as severe combined immunodeficiency (SCID). Despite a global estimated incidence of approximately 1 in 58,000 live births [4], only a handful of SCID cases have been formally reported annually in Malaysia, a figure that is more likely reflective of a failure in early diagnosis rather than a true population rarity. The absence of a national NBS programme to detect such conditions may be a major attributing cause of late presentations and consequently fatal outcomes [5]. This was also surmised in a report on the Southeast Asia PID Principles of Care (PoC) Life Index [6]. Comparative regional metrics show that Malaysia uniquely possesses full baseline availability of complex, advanced tertiary diagnostic modalities, yet its population-level PID diagnosis rate remains critically low, hovering between an estimated 1% and 10% of true theoretical prevalence [6]. Together, this underscores a paradox where advanced laboratory infrastructure exists, but the lack of universal frontline screening leaves up to 70% to 90% of affected infants globally and locally unrecognized until after severe clinical decline has occurred [6,7]. In aligning with recommendations by international expert bodies such as the W.H.O. and the Sustainable Developmental Goals (SDGs), specifically SDG 3 and SDG 10, the current state of NBS in Malaysia demands a systematic transition towards an expanded model.

This position statement, prepared by the Academy of Medicine of Malaysia College of Paediatrics (College), draws upon the latest clinical, economic, and bioethical evidence to present a comprehensive case for the expansion of Malaysia’s NBS programme. It addresses the specific conditions warranting prioritisation, the technological infrastructure required, the frameworks for equitable and sustainable financing, and the ethical obligations that underpin this public health imperative.

### The Wilson and Jungner Framework in NBS

The classic intellectual foundation for population-based NBS remains the criteria proposed by Wilson and Jungner in 1968 for the World Health Organization, which stipulate that a condition is appropriate for screening if (1) it is an important health problem; (2) there is an accepted treatment; (3) facilities for diagnosis and treatment are available; (4) there is a recognisable latent or early symptomatic stage; (5) there is a suitable test or examination; (6) the test is acceptable for the population; (7) the natural history of the condition is adequately understood; (8) there is an agreed policy on whom to treat; (9) the cost of case-finding is economically balanced in relation to possible expenditure on medical care as a whole; and (10) case-finding is a continuous process rather than a once-for-all project [8].

Every disorder discussed in this position statement satisfies these criteria. Universal NBS, implemented as a population-level public health programme, is further justified by the principle that such programmes are mandated only when risks are minimal, benefits are significant, and alternative approaches, such as clinical diagnosis post symptom onset, are incapable of achieving comparable outcomes [8]. The original Wilson and Jungner criteria were designed primarily for chronic disease screening in adults; the application of these criteria to neonatal inherited conditions, combined with the technological advances of the genomic era, has substantially expanded the range of conditions amenable to early population-based detection. Malaysia’s policy framework must evolve in concert with this evidence base.

## 2. The Clinical Imperative for Expansion

Currently, in Malaysia, umbilical cord blood collected immediately after delivery is used as the primary sample for universal NBS of G6PD deficiency and CH. Access to public healthcare is generally free to all citizens in Malaysia, including these tests provided at all government-funded facilities. Parallel to this is private healthcare, accessible to only a much smaller proportion of the population, where patients fully absorb the cost of care, usually paying out of pocket or with private insurance coverage. Expanded NBS has not yet been incorporated as a mandated programme in Malaysia’s public healthcare system. Recognising this gap, the Ministry of Health of Malaysia introduced the 2025 National Policy for Rare Diseases (NPRD), which highlighted the limitation of expanded NBS for inherited metabolic diseases, namely that it is currently only available upon request through selected public hospitals such as Universiti Malaya Medical Centre (UMMC) or in many private healthcare facilities [9]. Most of these facilities offer expanded NBS based on a variety panel of tests with different tiers in pricing. Prospective parents opt for those they prefer after being counselled on the tests, usually during antenatal clinic visits or parentcraft classes. Technologically, Malaysia has the capabilities and expertise to develop an expanded NBS programme. The Ministry of Health of Malaysia, through the Institute for Medical Research, has developed substantial expertise in biochemical genetics and rare disease diagnostics and the capabilities to detect multiple metabolic disorders from dried blood spot (DBS) specimens using tandem mass spectrometry (MS/MS). Even so, as current NBS utilises cord blood, heel-prick samples for DBS will require additional resources and training of personnel. One of the important strategic directions proposed in the NPRD is to establish regional centres covering the Central, Northern, Southern and East Coast regions of Peninsular Malaysia, as well as Sabah and Sarawak, to support expanded NBS and diagnostic confirmation of acute, life-threatening rare diseases. This regional model could reduce reliance on a centralised national referral laboratory and provide more equitable service support throughout the country. However, for expanded NBS to be made universal to most of the population who seek public healthcare, it first has to be adopted as a mandated public health programme so that the tests are offered at a minimal cost. Prioritising which additional tests to be rolled out in the expansion is another major area for consideration.

The expanded NBS programme relies on three core methods to detect congenital disorders. MS/MS is the cornerstone test for inherited metabolic conditions that test for the accumulated metabolites from biochemical pathway defects; immunoassays such as fluorometric and colorimetric assays detect enzyme or hormonal deficiencies; molecular and genetic testing have been used as secondary-tier or confirmatory assays for mutations of the genes causing the disease but are rapidly gaining traction now as a primary screening method, especially for SCID and spinal muscular atrophy (SMA).

### 2.1. Inborn Errors of Metabolism

IEM represents a prime target for expanded NBS. These disorders arise from biochemical defects in metabolic pathways. Affected infants are often asymptomatic in utero because the maternal circulation helps buffer the accumulation of toxic metabolites. However, once enteral feeding begins, these infants can deteriorate quickly, progressing to severe acidosis, hyperammonaemia, seizures, encephalopathy, and coma within hours to days. Without expanded NBS, the first presentation is typically a life-threatening metabolic crisis, by which point irreversible neurodevelopmental damage may have already occurred.

This clinical trajectory is not merely theoretical. A study examining IEM presentations within the Malaysian population revealed several barriers to early diagnosis, including limited clinician awareness of atypical and heterogeneous phenotypic expressions, which contributed to significant diagnostic delays. The study further identified a paucity of robust population-based epidemiological data on IEM, as well as a notable prevalence of multiple sibling involvement attributable to high rates of consanguinity. In response, the investigators highlighted the importance of careful family history recording and comprehensive genetic counselling as integral components of clinical management. Targeted screening of acutely ill infants and children in this cohort yielded a positivity rate of 2.0% (264 of 13,500 cases) [10], a scenario precisely aligned with what an expanded NBS programme is designed to prevent.

MS/MS applied to DBS samples collected on days two to three of life represents the gold standard for presymptomatic detection of a broad spectrum of IEM, including disorders of amino acid metabolism (such as phenylketonuria and maple syrup urine disease), organic acidurias (such as methylmalonic acidaemia and propionic acidaemia), and fatty acid oxidation disorders (such as medium-chain acyl-CoA dehydrogenase, or MCAD, deficiency). A single DBS sample analysed by MS/MS can simultaneously screen for 40 to 50 metabolic disorders, enabling a paradigm shift from the historical “one test, one metabolite, one disease” model to a “one test, many metabolites, many diseases” approach [11]. This has been demonstrated at scale across multiple continents, with evidence from Australia showing that the introduction of MS/MS-based NBS increased the detection rate of IEMs from approximately 8.6–9.5 per 100,000 births to 15.7 per 100,000 births (95% CI: 11.9–20.4), driven predominantly by the identification of previously undetected fatty acid oxidation disorders [12].

In the Malaysian context, a landmark pilot study conducted between 2006 and 2008, in which 30,247 newborns from 11 major public hospitals were screened for 27 IEMs usingMS/MS, identified a detection rate of 1 in 2916 newborns and confirmed cases including maple syrup urine disease, methylmalonic aciduria, argininosuccinic aciduria, ethylmalonic aciduria, and isovaleric aciduria [13]. The sensitivity of MS/MS was 80% and the specificity was 99%. The study explicitly concluded that “IEMs are common in Malaysia and NBS of IEM by MS/MS is a valuable preventive strategy by enabling the diagnosis and early treatment of IEM before the onset of symptoms”. It is noteworthy that the same investigators commented that “shortcomings warrant further solution so that in the near future NBS for IEM will become a standard of care for all babies in Malaysia in tandem with the developed world.” That study was conducted two decades ago, and the shortcomings, which were primarily tied to the fiscal policy at that time, remain unresolved today.

Progress has nonetheless been made. The UMMC initiated the first expanded NBS programme for IEMs in a Malaysian public hospital using MS/MS in 2015. In the absence of government-mandated IEM screening, parents either paid out of pocket or opted out if financially constrained. Under this model, 11,738 of 32,499 newborns (36.1% uptake) were screened using DBS obtained from heel pricks at 24 h of life (or after at least three full feeds) in the postnatal wards, resulting in the diagnosis of IEM in four patients [14]. More recently, a single-centre study in a Malaysian tertiary-level university teaching hospital screened over 4000 umbilical cord blood (UCB) samples collected on DBS cards, detecting one case of Pompe disease with a novel variant, alongside several cases with pseudo-deficient alleles [15]. Collaboration with an established overseas centre on this project has highlighted the success of international knowledge transfer in training and infrastructure expansion, key elements that could inform the development of a nationwide UCB-based screening programme and ultimately steer the implementation of expanded NBS.

Beyond clinical efficacy, the economic case for MS/MS-based screening is compelling. Early dietary modification (for conditions such as phenylketonuria andmaple syrup urine disease) or pharmacological intervention (for conditions such as isovaleric acidaemia) is substantially less costly than managing the consequences of recurrent acute metabolic crises, prolonged intensive care admissions, acquired intellectual disability, and lifelong disability support [16]. Health technology assessment frameworks from multiple countries have demonstrated favourable cost-effectiveness ratios for MS/MS-based expanded NBS, even in resource-constrained settings [17].

### 2.2. Inborn Errors of Immunity

#### SCID and X-Linked Agammaglobulinaemia

SCID is a group of rare but uniformly lethal genetic disorders characterised by absent or severely impaired T-lymphocyte production, resulting in profound susceptibility to all categories of infection. Without definitive treatment, SCID is fatal, typically from overwhelming infection, within the first year of life. The condition fulfils every Wilson and Jungner criteria for universal NBS, and the evidence for the cost-effectiveness and clinical benefit of population-based screening is now unequivocal.

The diagnostic cornerstone of NBS for SCID is the molecular quantitative PCR assay for T-cell receptor excision circle (TREC), which quantifies circular DNA fragments formed as a by-product of T-cell receptor recombination during thymic T-cell development [18]. In affected infants, TREC levels are markedly reduced or absent, reflecting inadequate thymic T-cell production. The TREC assay, performed on standard DBS cards, achieves close to 100% sensitivity for the detection of SCID [19]. Importantly, the assay simultaneously identifies a broad range of other T-cell lymphopenias, including DiGeorge syndrome, ataxia-telangiectasia, and Down syndrome with T-cell lymphopenia [20]. Furthermore, early diagnosis of SCID and treatment with allogeneic haematopoietic stem cell transplantation (HSCT) or, in selected cases, gene therapy yields survival rates exceeding 90% when intervention is initiated in the presymptomatic state, before the child has experienced recurrent, tissue-damaging infections [21]. In contrast, survival rates among children diagnosed only after symptomatic presentation are substantially lower, as pre-existing infections compromise HSCT outcomes.

Beyond TREC, there is clear clinical utility of the combined TREC and KREC (Kappa-deleting Recombination Excision Circle) assay [22]. While TREC remains the standard for SCID, adding KREC allows for the simultaneous detection of X-linked agammaglobulinaemia (XLA) and other primary immunodeficiencies characterised by B-cell lymphopenia, significantly enhancing the diagnostic yield on the same dried blood spot (DBS) samples without increasing procedural complexity. Incorporating the KREC biomarker into this multiplex configuration directly facilitates the presymptomatic identification of XLA [21]. In detecting B-cell lymphopenia at birth, clinical teams may pre-emptively institute immunoglobulin replacement therapy and prophylactic antibiotics [22]. This prevents the onset of recurrent, tissue-damaging sinopulmonary infections and significantly alters the survival trajectory of XLA infants, maximizing the diagnostic yield from a DBS test.

Malaysia’s situation is starkly illustrative of the consequences of the absence of NBS for SCID. A 2021 review documented that in the absence of a national NBS programme, “SCID patients typically present with natural progression of the disease and manifest with severe, recurring, or persistent infections, as well as opportunistic infections, within the first few months” [20]. The same review confirmed the availability of HSCT and demonstrated admirable success rates in Malaysian tertiary institutions, whilst also acknowledging that delayed diagnosis, delayed treatment initiation, and financial constraints for HSCT represent the primary barriers to optimal outcomes. NBS for SCID is thus not merely a screening test because it is the essential prerequisite for curative treatment to reach the patients who need it in time. To ensure screening results translate effectively into patient survival, standardized national clinical pathways should stipulate a ready and immediate link between the laboratory and specialized care [22]. For example, for successful local implementation, it mandates that an abnormal TREC/KREC result signal will trigger an automated, high-priority referral loop directly to the clinical immunologist [22,23]. This pathway bypasses the potentially long outpatient clinic waiting times for registration; rather, it could fast-track the patient to immediate protective isolation, accelerate direct confirmatory testing (flow cytometry and rapid genetics), facilitate deferral of future live-attenuated vaccines, and commence efforts to mitigate the risk of disseminated vaccine strain infections from at-birth-administered live vaccines such as BCG [22,24].

From a health economics perspective, a 2025 systematic review of eight model-based economic evaluations across the USA, Netherlands, Australia, UK, and New Zealand concluded that universal NBS for SCID is highly likely to be cost-effective from both health system and societal perspectives, with incremental cost-effectiveness ratios per quality-adjusted life-year gained ranging from USD 30,214 to USD 54,282 (2022 values) [25]. Thus, incorporating TREC testing into expanded NBS represents a manageable incremental investment with extraordinary potential returns.

### 2.3. Inherited Enzyme Hormonal and Genetic Defects

#### 2.3.1. Congenital Adrenal Hyperplasia

Congenital Adrenal Hyperplasia (CAH), primarily due to 21-hydroxylase deficiency, which accounts for over 90% of cases, is an autosomal recessive endocrinopathy characterised by impaired cortisol synthesis, excess ACTH drive, and androgen overproduction. The classical form of CAH due to severe enzyme deficiency is a life-threatening disease presenting in the early newborn period. Affected neonates develop electrolyte crises (hyponatraemia, hyperkalaemia), cardiovascular collapse, and can die within the first two weeks of life if unidentified and untreated. These patients belong to the salt-wasting form, which accounts for approximately 75% of classical CAH cases. The remaining 25% of cases comprise the simple virilising form, presenting as ambiguous genitalia, which is also a paediatric emergency. Early CAH treatment prevents not only acute adrenal crises and death, but also incorrect sex assignment in affected females, psychosocial sequelae, and long-term impairment of growth and fertility.

The relevance of these clinical risks to Malaysia is direct, well documented and of high impact. The incidence of classic CAH in Malaysia is estimated to be approximately 1:3000 births. This prevalence is much higher than the global average (typically 1:14,000) and is attributed to local cultural norms, such as consanguineous marriages, resulting in genetic aberrations [26]. Furthermore, a 2024 study found that Malaysian neonates had 29.3 times higher odds of CAH diagnosis compared to infants and children, confirming the preponderance of early-onset disease [27]. The authors explicitly state that “despite [the NBS screening for CAH] not being available in Malaysia, screening for CAH has been incorporated as part of an extensive NBS programme worldwide and has been shown to be successful in lowering the incidence of fatal salt-wasting crises and preventing delayed or misdiagnosis”.

Implementing a nationwide NBS protocol requires a robust, two-tiered diagnostic and clinical response framework to handle the disease’s aggressive timeline. Standard NBS for CAH involves the baseline measurement of 17-hydroxyprogesterone (17-OHP) from DBS samples, with secondary confirmatory testing using liquid chromatography–tandem mass spectrometry (LC-MS/MS) steroid profiling to minimise false positives. Because of the rapid onset of salt-wasting symptoms, screening infrastructure must link immediately to clinical intervention. Reflecting this urgency, the Australia and New Zealand Society for Paediatric Endocrinology and Diabetes has published guidance confirming that screen-positive infants are at high risk of deterioration if treatment is delayed while confirmatory tests are awaited and recommends urgent clinical assessment and empirical treatment on the day of recall [28].

Given this established clinical pathway, the omission of CAH from Malaysia’s national NBS panel is particularly difficult to justify. The initial DBS screening test is relatively low-cost, the condition is thoroughly characterised, and the mandatory maintenance treatment, hydrocortisone and fludrocortisone replacement therapy, is inexpensive, effective, and already widely available within the Malaysian public healthcare system. Transitioning CAH screening from a gap in care to a standardized nationwide programme presents a highly cost-effective opportunity to eliminate preventable neonatal mortality and secure long-term health outcomes for affected children.

#### 2.3.2. Spinal Muscular Atrophy

If any single condition exemplifies the transformative power of early detection in the genomic era, it is 5q-linked SMA. Spinal muscular atrophy is an autosomal recessive neuromuscular disorder caused by biallelic deletion of the survival motor neuron 1 (SMN1) gene, resulting in progressive motor neuron degeneration. Historically, SMA type I, the most severe and most common genotype, was characterised by the onset of symptoms before six months of age, progressive respiratory failure, and invariable death before the second birthday. No effective treatment existed.

The landscape has been fundamentally transformed. Three disease-modifying therapies are now approved: nusinersen (Spinraza^®^; Biogen Inc., Cambridge, MA, USA), an intrathecal antisense oligonucleotide targeting SMN2 pre-mRNA splicing; onasemnogene abeparvovec (Zolgensma^®^; Novartis Pharma AG, Basel, Switzerland), a single-dose intravenous gene replacement therapy delivering a functional SMN1 copy; and risdiplam (Evrysdi^®^; F. Hoffmann-La Roche Ltd., Basel, Switzerland), an oral small-molecule SMN2 splicing modifier [29]. All three have demonstrated disease modification, but the magnitude of benefit is profoundly dependent on the timing of intervention relative to symptom onset.

The evidence base is unambiguous. The NURTURE trial enrolled presymptomatic infants (less than six weeks of age at first dose) with confirmed SMN1 biallelic deletion and followed them with nusinersen. At interim analysis with a median follow-up of 2.9 years, all 25 participants were alive, none required tracheostomy or permanent ventilation, and 88% achieved the ability to walk independently, a milestone essentially unheard of in untreated SMA type I [30]. The SPR1NT trial of onasemnogene abeparvovec in presymptomatic infants with two SMN2 copies showed that all 14 enrolled infants achieved gross and fine motor scores similar to unaffected children at least once during the study, and critically, none required any form of mechanical feeding or respiratory support, compared to 32% of symptomatic patients who required feeding support and 18% who required ventilatory support by 18 months of age in the historical comparison arm [31]. The RAINBOWFISH trial of risdiplamsimilarly demonstrated substantial preservation of motor function when initiated before symptom onset [32].

The mechanistic basis for this timing dependency is well established: SMA has a strong developmental component in which the SMN protein is crucial for motor neuron development, and irreversible loss of these neurons occurs early in life. NBS for SMA, using real-time quantitative PCR (RT-qPCR) to detect homozygous deletion of exon 7 of the SMN1 gene from DBS samples, is technically reliable and well validated [30]. As of 2023, NBS programmes for SMA operate in 33 countries across Europe, North America, Australasia, Taiwan, and beyond, with approximately 62 countries having made disease-modifying therapies available [33]. Malaysia’s continued absence from this list translates directly into preventable deaths and preventable disability for an entirely identifiable and treatable population.

Encouragingly, local expertise in administering these targeted therapies has already been established. The UMMC initiated the first gene replacement therapy in Malaysia in 2020 for SMA type 1 with onasemnogene abeparvovec, following its approval by the U.S. Food and Drug Administration in May 2019. The initial experience with the first four Malaysian patients with SMA type 1, who were symptomatic and received compassionate treatment, wasreported, along with the ethical challenges posed by the treatment’s cost of USD9 million per patient. Critically, these patients were diagnosed late in infancy and were not detected through expanded NBS [34]. Subsequently, an additional four SMA type 1 patients received gene therapy through funds raised via crowdfunding sources.

This experience highlights a paradox: Malaysia possesses the clinical capacity to deliver advanced gene therapies yet lacks the screening infrastructure to identify candidates before symptom onset. Expanded NBS for SMA would enable early, presymptomatic intervention, yielding substantially more favourable outcomes, as the NURTURE, SPR1NT, and RAINBOWFISH trials have definitively shown, and would simultaneously obviate the need for the ethically fraught and burdensome crowdfunding efforts currently relied upon to treat late-diagnosed children.

### 2.4. Inclusion of Emerging and Regionally More Common Disorders

Global NBS standards have long included screening for a broad range of conditions for which early management demonstrably alters outcome. Many of these disorders present clinically as jaundice in the early neonatal period, a presentation of relevance in Asia, where neonatal hyperbilirubinemia is significantly more common. Infants of East Asian parentage have 37% increased risks of developing jaundice and a 70% increased risk of requiring phototherapy or blood transfusion compared to their white counterparts [35]. This epidemiological reality has historically driven Malaysia’s leadership in universal NBS for G6PD deficiency, initiated in 1980, precisely because of the high population incidence and morbidity from severe hyperbilirubinemia secondary to this enzyme defect. CH was subsequently included in Malaysia’s universal NBS programme after the year 2000.

Beyond G6PD deficiency, emerging genetic variants associated with severe and prolonged hyperbilirubinemia, notably those in the UGT1A1 and SLC10A1 genes [36], warrant serious consideration for inclusion in an expanded NBS programme in Asia. Detection of these conditions would enable genetic counselling and prepare parents for the possibility of repeated and protracted hospitalisations for phototherapy, thereby preventing complications arising from bilirubin-induced neurological dysfunction. Bilirubin toxicity remains a major cause of severe childhood disability, including deafness and cerebral palsy, yet these outcomes are largely preventable with simple, cost-effective phototherapy.

Several enzymatic and genetic defects presenting as early or prolonged neonatal jaundice can be detected through a dedicated “jaundice panel” or as individual components of an expanded NBS programme. One well-established IEM already included in routine NBS in many countries is galactosaemia, caused by a deficiency of galactose-1-phosphate uridylyltransferase. This condition presents in the neonatal period with jaundice, hepatic dysfunction, cataracts, and life-threatening *E. coli* sepsis; dietary exclusion of galactose from milk feeds, initiated within days of birth, prevents most acute manifestations. More recently, a genetic “jaundice panel” based on next-generation sequencing has been introduced, testing five genes (*ABCB11*, *ABCB4*, *ATP8B1*, *JAG1*, and *TJP2*) that encompass a group of disorders causing intrahepatic cholestasis, which presents as neonatal jaundice and can lead to persistent liver dysfunction in childhood [37].

The inclusion of conditions common in Asian newborns within such a panel is essential. For example, a Malaysian study reported that citrin deficiency, associated with the *SLC25A13* gene, may present in infants with life-threatening cholestasis, often mistaken for biliary atresia [38], findings subsequently confirmed by numerous other reports from the region [39,40].

Regional peers and global standards have long substantiated the case for expansion. In Singapore, KK Women’s and Children’s Hospital has operated their national expanded NBS programme screening for more than 40 inheritable metabolic disorders since 2019 [41]. Taiwan operates a comprehensive programme for 22 congenital disorders, including SMA, with extensive outcome data demonstrating reduced mortality, morbidity, and long-term disability, and has achieved nearly 100% screening coverage nationwide [42]. Japan currently screens for 20 diseases at a national level with 100% acceptance rates and world-class quality control systems [43]. Thailand offers free NBS for 24 rare conditions to all newborns within 24 h of birth, with the government covering treatment costs [44]. The Philippines has recently expanded to 29 screening conditions, drawing on experiences from U.S. programmes and data from California on Filipinos born and screened there [45]. A detailed summary of the current status and conditions screened in some countries in this region is published by Therrell BL et al. [1].

Malaysia should align with international standards and mirror the strategies adopted towards successful implementation of the expanded NBS by these regional neighbours. Most of the available technologies and the clinical expertise required to implement expanded NBS already exist within Malaysian tertiary healthcare institutions. The barrier to implementation is not the scientific capacity but more of political will and resource allocation priorities.

### 2.5. Call for Action—A Proposed Strategy to Expand NBS in Malaysia

For Malaysia, expanded NBS represents an opportunity to move rare disease care from a predominantly reactive model—diagnosing children after they become ill—to a proactive model of early detection and prevention of disability. Importantly, the addition of tests in NBS should not be viewed simply as expanding the array of laboratory tests. It should form part of an integrated “screen–confirm–counsel–treat–follow-up” pathway. Positive screening results must be followed expediently by confirmatory testing, specialist assessment, genetic counselling, treatment and long-term monitoring.

The way forward for Malaysia perhaps lies in a multi-phased NBS expansion strategy. Building upon the existing infrastructure and setup may be the reasonable pragmatic approach. Currently, UCB is collected to screen for G6PD deficiency and CH. This method of sampling is convenient, readily accessible and painless. A major advantage is that samples are obtained even from newborn infants who are discharged early from the hospital in less than 24 h, who make up a significant proportion of cases in this country. While UCB may have some limitations such as potential maternal blood contamination and insufficient endogenous metabolite accumulation, several studies have reported its validity for the detection of non-metabolite-based conditions [15,46,47]. Malaysia could begin by establishing a nationally agreed core panel of conditions as additional screening tests using UCB, recommended by a taskforce steered by a specialist advisory committee. These conditions should be of a significant health burden, detectable with reliable screening and confirmatory tests, and have effective intervention after diagnosis. Three conditions top the list: CAH, SCID and SMA as discussed earlier, benefit from available expertise and the appropriate support to manage affected individuals. Programme expansion can be phased out progressively, hospital by hospital, state by state as laboratory capacity and treatment availability increases.

Nevertheless, the impact of many inherited metabolic diseases benefitting from expanded NBS underscores the life-threatening consequences of delayed diagnosis. Heel-prick DBS is currently the sampling method of choice to screen these metabolite-based conditions. The local landmark pilot study to expand NBS conducted two decades ago showed the resounding success and feasibility of this approach. Although many public hospitals practise early discharge policy after delivery, Malaysia has a reliable network of community healthcare support to monitor postpartum mothers and newborn infants after hospital discharge. The infants are examined primarily for jaundice during each visit. These healthcare providers could be trained to concurrently collect heel-prick DBS samples at the visit for laboratory testing. The sample assay method chosen would preferably leverage previous local experience using MS/MS. Some countries, however, adopt a genomic testing basis in their nationwide expanded NBS programme to overcome the significant lag time from test validation and improve diagnostic yield over a traditional biochemical screening model [48]. The combination of this next phase of expanded NBS using heel-prick DBS, the selection of the additional tests and the preferred assay method of choice will rely on expert guidance from an appointed expert advisory committee.

## 3. Ethical Considerations

### 3.1. Justice, Equity, and the Child’s Right to an Open Future

The ethics of NBS extends beyond clinical utility to a set of moral principles that bear directly upon Malaysia’s obligations to its youngest citizens. The philosophical concept of the “child’s right to an open future,” articulated by Joel Feinberg [49] and subsequently applied extensively within paediatric bioethics, holds that decisions made on behalf of children should preserve, rather than foreclose, their future autonomy and life opportunities [50]. This principle generates a clear and powerful normative demand. When an effective treatment exists for a condition that is identifiable in the neonatal period, the deliberate non-implementation of screening constitutes an arbitrary limitation of the child’s prospects for health and full participation in life.

The principle of distributive justice further demands that the benefits of NBS be available universally, irrespective of geographic location, socioeconomic status, or ethnicity. In a healthcare system that already provides G6PD and CH screening to all newborns at no personal cost, the selective denial of equally well-evidenced screening for SCID, CAH, and metabolic disorders creates an unjustifiable two-tier system of neonatal care. Children born to families with the financial means to access private expanded NBS panels, now offered commercially in Malaysia, receive detection and early treatment that their peers from lower-income households do not. This disparity is morally indefensible and directly contradicts Malaysia’s constitutional commitments to equal treatment and Article 4 of the UN Convention on the Rights of the Child, which requires states to undertake measures to ensure the realisation of children’s right to the highest attainable standard of health [51].

### 3.2. Utilitarian Justifications and the Ethics of Omission

A utilitarian framing of NBS policy yields equally forceful conclusions. The collective suffering avoided by early detection and treatment of one case of SMA type I, one case of SCID, or one case of salt-wasting CAH substantially exceeds the marginal cost of screening the entire birth cohort. When this calculation is performed across a national birth cohort of approximately 500,000 live births annually in Malaysia, and when the lifetime costs of disability, institutional care, and caregiver burden are incorporated, the investment in expanded NBS generates positive net societal returns by any standard economic methodology.

Critically, the literature strongly supports the position that omission in NBS is not ethically neutral: failing to screen constitutes a decision with predictable negative outcomes for identifiable children [52]. The rule of rescue provides a compelling justification for mandatory screening when effective interventions exist but also raises tensions around autonomy and consent—especially as programmes expand into genomics or untreatable conditions [53,54]. While utilitarian perspectives may downplay distinctions between acts and omissions [55], public sentiment often views omissions as less blameworthy, a bias challenged by ethicists advocating proactive intervention when harm can be prevented.

Quality evidence supports mandatory screening for high-benefit conditions due to clear reductions in morbidity/mortality from early detection [56], but evidence becomes less robust as panels expand or uncertainty increases (e.g., genomic findings without clear treatment pathways) [57]. Psychosocial harms from false positives are real but generally transient if communication is managed well. However, these risks must be weighed against the severe consequences of missed diagnoses [58].

### 3.3. Consent, Transparency, and Culturally Responsive Communication

Whilst the ethical arguments for universal expanded NBS are compelling, their implementation must be accompanied by robust frameworks for consent, transparency, and community engagement. These considerations become salient when NBS employs a primary genomic testing approach. The complexity of interpreting and managing newly identified variants, variants of unknown significance, and conditions with variable phenotypic expression (such as leaky SCID) presents significant challenges. This complexity is further amplified by the use of high-throughput molecular tools, which inevitably uncover incidental and non-target variants [59]. Multiplex screening may also unravel conditions such as variant or “leaky” immunodeficiencies, secondary lymphopenias, or syndromes not amenable to treatment, such as ataxia-telangiectasia [59]. Such findings risk the premature medicalisation of an otherwise asymptomatic infant and may create a distinct tension between clinical utility and parental psychological distress, particularly when identifying conditions with highly variable phenotypic outcomes.

These realities justify a shift away from exhaustive, target-by-target consent documents toward a structured “process consent” model. This approach encompasses continuous prenatal parental education, while anchoring disclosure within a clear, actionable health management framework [59]. It acknowledges that informed consent is not a one-time event but an ongoing dialogue that evolves alongside the child’s development and the emergence of new clinical information.

Beyond the interpretive challenges of genomic data, the economic and procedural dimensions of treatment must also be considered. The cost of therapy may be prohibitive for many families, and treatment options themselves carry procedural risks, with HSCT for SCID and gene therapy for SMA being notable examples.

Equally critical is the development of public education programmes that are linguistically accessible, culturally attuned to Malaysia’s diverse population, and sensitive to the religious and cultural dimensions surrounding genetic testing. In Malaysia’s multicultural society, community engagement strategies for expanded NBS must be participatory and iterative, actively involving stakeholders from various ethnic and religious backgrounds in their design and refinement. Parental information materials should distinguish between disorders with high penetrance and straightforward treatment algorithms, such as CAH and metabolic disorders amenable to dietary restriction, and those with more complex treatment pathways, where outcomes may be less predictable and management more demanding. Such differentiation empowers parents to make truly informed decisions while mitigating unnecessary anxiety.

### 3.4. Intergenerational Justice and the Social Contract

The concept of intergenerational justice or the obligation of present generations to make decisions that protect the opportunities and wellbeing of future generations provides an additional ethical framework for Malaysia’s NBS policy. A society that possesses the technical capacity to prevent a child from dying of SCID, developing profound intellectual disability from a metabolic disorder, or losing all motor function from SMA, but that chooses not to deploy that capacity, fails a fundamental test of civic obligation. The investment in NBS infrastructure made today produces health dividends extending across the lifetimes of the children screened, decades of productive, healthy life that would otherwise be truncated or severely limited.

This framing is particularly pertinent in the Malaysian context, where the Ministry of Health’s Health White Paper articulates a strategic shift toward a more preventive, sustainable, and technology-driven health ecosystem [60]. By prioritising early interventions and resource optimization, the Health White Paper provides the policy support necessary to transition from a curative-centric model to one that emphasises long-term population health outcomes. Indeed, the Health White Paper showed that infant mortality rates in Malaysia have remained relatively unchanged since 2000. The first and second most common preventable underlying cause of deaths for children under five were conditions originating in the perinatal period (32.3%), followed by congenital disorders (19.2%) [61]. Integrating universal expanded NBS into this reformed health architecture is a direct operationalisation of the White Paper’s mandate to reduce the country’s infant mortality rate, modernise service delivery, ensure health equity, and safeguard the wellbeing of future generations.

## 4. Innovative and Sustainable Financing Mechanisms

The most cited barrier to expanded NBS in Malaysia is fiscal. The College rejects the premise that expanded NBS is unaffordable. On the contrary, the appropriate question is not whether Malaysia can afford expanded NBS, but whether it can afford the far greater long-term costs of not implementing it. The hidden costs of untreated IEM, comprising recurrent acute hospital admissions, intensive care unit stays, long-term institutional care, special education provision, lost caregiver productivity, and lifetime disability support, dwarf the cost of universal expanded NBS implementation [16]. Nonetheless, sustainable and diversified financing mechanisms are necessary to insulate the NBS programme from cyclical health budget pressures.

One such effort in that direction is the SHINE (Screening for Health, Intervention and Nurturing of Every child) initiative as the national framework for operationalising universal expanded NBS [22]. SHINE serves as a strategic model to foster public–private partnerships and sustainable financing, with the vision of establishing Malaysia as both a national and international reference centre for expanded NBS in Southeast Asia. An analytical review of the SHINE configuration demonstrates that its utility extends beyond a simple fiscal framework; it functions as a highly integrated operational and laboratory ecosystem [22]. By establishing a centralised, high-throughput laboratory referral network, SHINE bridges the gap between initial DBS collection at primary health clinics and tertiary-level care centres. The model relies on multiplexed technology platforms (such as combined TREC/KREC/SMN1 configurations) to optimise cost-effectiveness, integrates data management directly with the Malaysian National Health Data Warehouse, and applies rigorous external quality assurance to build a dependable, scalable public health infrastructure [22,24].

## 5. Governance, Quality Assurance, and Advisory Oversight

The establishment of a dedicated National NBS Advisory Committee (NNSAC) is essential to provide ongoing scientific and policy stewardship. The NNSAC should be a multidisciplinary, multipartite body comprising paediatricians (including metabolic disease and genetic specialists), laboratory scientists, neonatologists, paediatric immunologists, neurologists, and endocrinologists, alongside bioethicists, patient and family advocates, Ministry of Health representatives, and health economists. Its remit should include (a) annual review and evidence-based updating of the national NBS panel; (b) quality assurance oversight including laboratory external quality assurance participation; (c) coordination of follow-up pathways and specialist referral networks; (d) monitoring of programme outcomes; and (e) public reporting of NBS programme performance metrics.

A linked national NBS registry, integrated with the Malaysian National Health Data Warehouse, should be established to capture clinical outcomes for all screen-positive infants, enabling continuous programme evaluation and the generation of the long-term Malaysian-specific outcome data needed to refine health economic models over time.

## 6. Addressing Anticipated Objections

### 6.1. The Costs of Expanded NBS and Its Downstream Therapies Are Prohibitive

The costs of not screening are demonstrably greater. A single case of SMA type I managed palliatively with repeated hospitalisation, respiratory support, nutritional interventions, and eventual end-of-life care incurs substantial direct and indirect costs, whilst producing none of the health gains achievable with presymptomatic treatment. Economic analyses from multiple countries consistently demonstrate that NBS programmes generate positive net economic benefit over a lifetime horizon [25]. The appropriate policy response is to negotiate sustainable access to high-cost therapies through managed access frameworks, not to withhold screening.

### 6.2. Malaysia Lacks the Laboratory Infrastructure for Expanded NBS

The pilot MS/MS study in 2006–2008 demonstrated that Malaysian public hospital laboratories could operate MS/MS-based IEM screening at scale [13]. Enzymatic assays, such as 17-OHP for CAH, TREC assays for SCID, and SMA qPCR, can be performed as additional tests alongside CH and G6PD screening. A phased approach, beginning with established reference laboratories such as the UMMC and Malaysia’s Institute for Medical Research, and extending progressively to regional centres, is operationally feasible and consistent with how other regional NBS programmes have scaled.

### 6.3. Cultural and Religious Concerns About Genetic Testing May Impede Uptake

Experience from Muslim-majority countries, including Iran, Turkey, and Saudi Arabia, demonstrates that properly designed, culturally sensitive NBS programmes achieve high uptake and community acceptance when framed around the actionable health benefits of early identification, rather than the genetic determinism that may carry cultural stigma [62,63,64,65]. Community engagement, multilingual educational materials, and the involvement of religious and community leaders in programme design are essential components of any implementation strategy for Malaysia.

## 7. The Role of the Academy of Medicine of Malaysia

The Academy of Medicine of Malaysia has a responsibility as the premier multi-specialty medical college in Malaysia to provide authoritative, evidence-based policy guidance on matters of national health significance. The Academy’s related disciplines collectively possess the expertise to drive the scientific case for expanded NBS, to participate in the governance of the proposed NNSAC, to develop clinical pathways and follow-up protocols for screen-positive infants, and to contribute to the training of the expanded clinical workforce required to manage the increased caseload.

The College therefore exercises here with due diligence to state its position statement to the governing bodies for an urgent review of the national NBS programme and to establish a high-level multidisciplinary working group to develop a national expanded NBS roadmap with defined timelines, measurable milestones, and accountable implementation mechanisms.

Malaysia stands at a critical juncture in the evolution of its public health commitment to child welfare. The historical NBS programme, encompassing G6PD deficiency and CH, has delivered foundational benefits and represents a genuine public health success. But the medical landscape has transformed very much. Conditions that were once untreatable are now curable, provided treatment reaches the child in the narrow presymptomatic window. Diseases that once guaranteed early death or lifelong disability, SMA type I, SCID, salt-wasting CAH, and the broad spectrum of IEMs, are now manageable with outcomes approaching normality, but only with early identification through systematic screening. The economic evidence demonstrates cost-effectiveness across all priority conditions. The ethical framework grounded in distributive justice, the child’s right to an open future, intergenerational equity, and the moral weight of preventable omission demands action. The imperative is clear, the evidence is available, and the time for action is long overdue.

The College recommends that expanding NBS adopts a model of process consent and parental education, rather than elaborate individualised informed consent for each condition, consistent with international best practice for population-based public health screening programmes [8]. The emphasis should be placed on the actionable nature of results, the benefits of early identification, and the safeguards in place for result communication and follow-up.

In addressing the fiscal aspect viewed as the major hurdle in an expanded NBS programme, the College proposes a three-tiered financing strategy:

- Hypothecated Health Taxation: The establishment of a dedicated National NBS Fund via hypothecation or the earmarking of revenue from health-focused excise duties (specifically levies on tobacco, alcohol, and sugar-sweetened beverages). This fiscal instrument creates a justifiable link between the negatives of unhealthy consumption and the positives of paediatric preventive health.
- Managed Entry Agreements and Value-Based Procurement: For rare diseases necessitating ultra-expensive interventions (e.g., enzyme replacement therapies, gene therapies for SMA), the College advocates Managed Entry Agreements with pharmaceutical stakeholders. These partnerships should transcend traditional procurement, focusing instead on “value-based” models where industry participation is contingent upon the efficacy of early diagnosis.
- Implementation Science and Capacity-Building Grants: While operational costs must remain a sovereign responsibility, initial capital expenditure, specifically for the pilot and scale-up phases, can be supported through international research grants and global health partnerships. These funds are designated for implementation science: developing local protocols, training clinical and laboratory staff, and establishing centralised data registries.

## Data Availability

No new data were created or analyzed in this study. Data sharing is not applicable to this article.
